# Machine-learning scoring functions for identifying native poses of ligands docked to known and novel proteins

**DOI:** 10.1186/1471-2105-16-S6-S3

**Published:** 2015-04-17

**Authors:** Hossam M Ashtawy, Nihar R Mahapatra

**Affiliations:** 1 Department of Electrical and Computer Engineering, Michigan State University, East Lansing, Michigan 48824, USA

**Keywords:** docking power, ligand pose identification, machine learning, molecular docking, scoring functions

## Abstract

**Background:**

Molecular docking is a widely-employed method in structure-based drug design. An essential component of molecular docking programs is a scoring function (SF) that can be used to identify the most stable binding pose of a ligand, when bound to a receptor protein, from among a large set of candidate poses. Despite intense efforts in developing conventional SFs, which are either force-field based, knowledge-based, or empirical, their limited docking power (or ability to successfully identify the correct pose) has been a major impediment to cost-effective drug discovery. Therefore, in this work, we explore a range of novel SFs employing different machine-learning (ML) approaches in conjunction with physicochemical and geometrical features characterizing protein-ligand complexes to predict the native or near-native pose of a ligand docked to a receptor protein's binding site. We assess the docking accuracies of these new ML SFs as well as those of conventional SFs in the context of the 2007 PDBbind benchmark dataset on both diverse and homogeneous (protein-family-specific) test sets. Further, we perform a systematic analysis of the performance of the proposed SFs in identifying native poses of ligands that are docked to novel protein targets.

**Results and conclusion:**

We find that the best performing ML SF has a success rate of 80% in identifying poses that are within 1 Å root-mean-square deviation from the native poses of 65 different protein families. This is in comparison to a success rate of only 70% achieved by the best conventional SF, ASP, employed in the commercial docking software GOLD. In addition, the proposed ML SFs perform better on novel proteins that they were never trained on before. We also observed steady gains in the performance of these scoring functions as the training set size and number of features were increased by considering more protein-ligand complexes and/or more computationally-generated poses for each complex.

## Introduction

### Background

**Figure 1:**
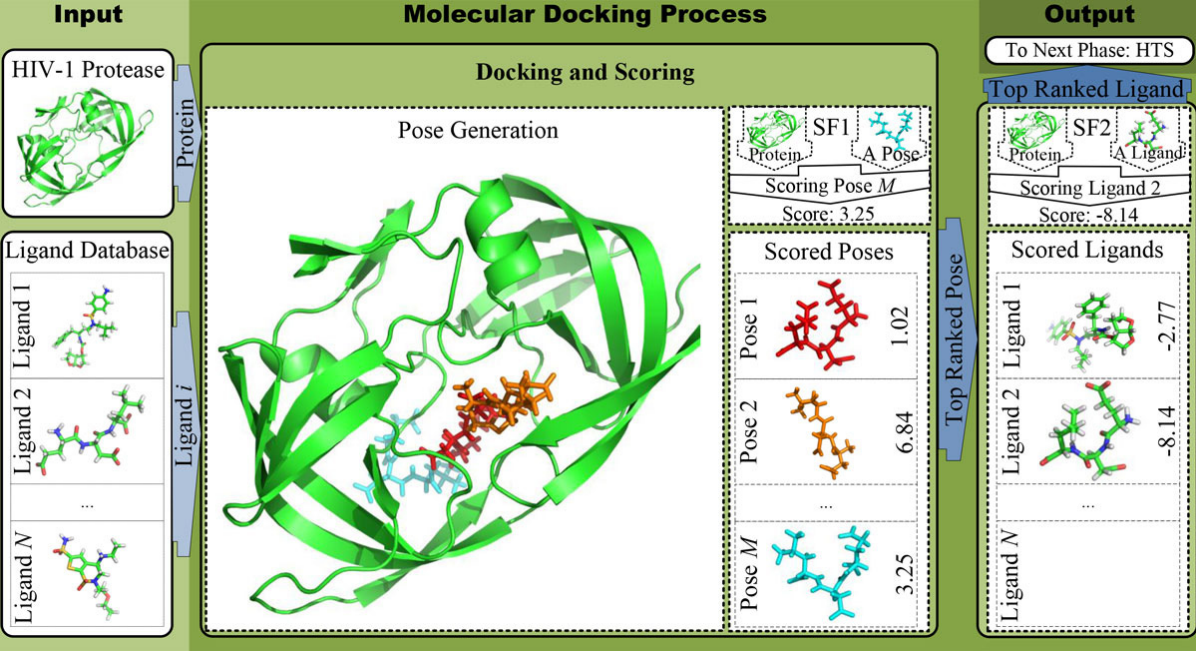
**Protein-ligand docking and ranking workflow**.

Bringing a new drug to market is a complex process that costs hundreds of millions of dollars and spans over ten years of research, development, and testing. A fairly big portion of this hefty budget and long time-line is spent in the early stages of drug design that involves two main steps: first, the enzyme, receptor, or other protein responsible for a disease of interest is identified; second, a small molecule or *ligand *is found or designed that will bind to the target protein, modulate its behavior, and provide therapeutic benefit to the patient. Typically, *high-throughput screening *(HTS) facilities with automated devices and robots are used to synthesize and screen ligands against a target protein. However, due to the large number of ligands that need to be screened, HTS is not fast and cost-effective enough as a lead identification method in the initial phases of drug discovery [1]. Therefore, computational methods referred to as *virtual screening *are employed to complement HTS by narrowing down the number of ligands to be physically screened. In virtual screening, information such as structure and physicochemical properties of a ligand, protein, or both, are used to estimate both *binding pose *and/or *binding affinity*, which represents the strength of association between the ligand and its receptor protein. The most popular approach to predicting the correct binding pose and binding affinity (BA) in virtual screening is *structure-based *in which physicochemical interactions between a ligand and receptor are deduced from the 3D structures of both molecules. This *in silico *method is also known as *protein-based *as opposed to the alternative approach, *ligand-based*, in which only ligands that are biochemically similar to the ones known to bind to the target are screened. In this work, our focus will be on protein-based drug design, wherein ligands are placed into the active site of the receptor. The 3D structure of a ligand, when bound to a protein, is known as *ligand active conformation. Binding mode *refers to the orientation of a ligand relative to the target and the protein-ligand conformation in the bound state. A binding pose is simply a candidate binding mode. In *molecular docking*, a large number of binding poses are computationally generated and then evaluated using a *scoring function (SF)*, which is a mathematical or predictive model that produces a score representing binding stability of the pose. The outcome of the docking run, therefore, is a ligand's top pose ranked according to its predicted binding score as shown in Figure 1. Typically, this docking and scoring step is performed iteratively over a database containing thousands to millions of ligand candidates. After predicting their binding poses, another scoring round is performed to rank ligands according to their predicted binding free energies. The top-ranked ligand, considered the most promising drug candidate, is synthesized and physically screened using HTS.

The most important steps in the docking process are scoring ligands' conformations at their respective binding sites and ranking ligands against each other. These core steps affect the outcome of the entire drug search campaign. That is because predictions of scoring functions determine which binding orientation/conformation is deemed the best, which ligand from a database is considered likely to be the most effective drug, and the estimated binding affinity (BA). Correspondingly, three main capabilities that a reliable scoring function should have are: (i) the ability to identify the correct binding mode of a ligand from among a set of (computationally-generated) poses, (ii) the ability to correctly rank a given set of ligands, with known binding modes when bound to the same protein, and, finally, (iii) the ability to produce binding scores that are (linearly) correlated to the experimentally-determined binding affinities of protein-ligand complexes with known 3D structures. These three performance attributes were referred to by Cheng et al. as *docking power, ranking power*, and *scoring power*, respectively [2]. We refer to the corresponding problems as *ligand docking, ligand ranking*, and *ligand scoring *problems. In practice and in all existing work, a single general SF is trained to predict protein-ligand BA and then used in both the ligand docking and ranking stages to identify the top pose and ligand, respectively. In this work, we propose docking-specialized machine-learning SFs capable of predicting native poses more accurately than the conventional BA-based SFs. These native-pose prediction models are used as SF1 in Figure 1. As for the second scoring round, designated by SF2 in Figure 1, in previous work we built accurate machine-learning SFs to score and rank ligands against each other using their predicted binding affinities [3,4].

### Related work

Most SFs in use today can be categorized as either *force-field-based *[5], *empirical *[6], or *knowledge-based *[7] SFs. Despite intense efforts into these conventional scoring schemes, several recent studies report that the docking power of existing SFs is quite limited. Cheng and co-workers recently conducted an extensive test of sixteen SFs from these three categories that are either employed in mainstream commercial docking tools and/or have been developed in academia [2]. The main test set used in their study consisted of 195 diverse protein-ligand complexes and four other protein-specific test sets from PDBbind 2007 database [8]. In order to assess the docking power of all SFs, they generated 100-pose decoy sets for each protein-ligand complex in the main test set. They defined the *docking power *of an SF as its rate of success in identifying binding poses that are within a certain root-mean-square deviation (RMSD) from the native pose over all complexes. Using this criteria, three SFs were found to have a relatively higher level of accuracy when their docking abilities were judged in three different experiments. These SFs are ASP [9] in the GOLD [10] docking software, PLP1 [11] in Discovery Studio [12], and the stand-alone SF DrugScore [13]. In a follow-up study [14], Plewczynski et al. evaluated the docking performance of seven popular docking programs (Surflex, LigandFit, Glide, GOLD, FlexX, eHiTS, and AutoDock) on a larger data set composed of 1300 protein-ligand complexes which constitute the refined set of PDBbind 2007. They measured the accuracies of these programs in both pose prediction and scoring capabilities and found that there was no single docking tool that consistently outperformed all others. GOLD and eHiTS achieved the highest docking accuracy of 60% in terms of the percentage of complexes whose top score conformations are within 2Å from the native poses. The team also found that there is a very weak correlation between predicted docking scores and measured binding affinities. Similar findings were published recently by Yamaotsu et al. [15] suggesting that the docking accuracies of GOLD, eHiTS and FRED were better than those of AutoDock, AutoDock Vina, and DOCK.

In this work, we will compare our novel ML SFs against the sixteen conventional SFs considered by Cheng et al. [2]. They used the four popular docking programs LigandFit [16], GOLD, Surflex [17], and FlexX [18] to generate diverse sets of decoy poses. Each of these tools employs different conformational search algorithms for best poses. Namely, LigandFit relies on a shape-directed algorithm, GOLD uses a genetic algorithm, Surflex is guided by a molecular-similarity based algorithm, and FlexX employs an incremental construction algorithm as a search engine [2]. They then combined the generated poses of each program and selected a subset of 100 decoys according to a systematic clustering procedure that will be explained later in more detail. The intention behind using four different docking algorithms was to explore the conformational space as thoroughly as possible and to avoid a potential sampling bias of this space if only one program were to be used.

In previous work, we have presented BA-based ML models for the ligand scoring and ranking problems [3,4]. However, the focus of this work is on the ligand docking problem and we present docking-specialized ML SFs in which we consider a more diverse collection of features and an explicit modeling of RMSD of binding poses, which dramatically improve docking performance.

### Key contributions

Various nonparametric ML methods inspired from statistical learning theory are examined in this work to model the unknown function that maps structural and physicochemical information of a protein-ligand complex to a corresponding distance to the native pose (in terms of RMSD value). Ours is the first work to perform a comprehensive assessment of the docking accuracies of conventional and machine-learning (ML) SFs across both diverse and homogeneous (protein-family-specific) test sets using a common diverse set of features across the ML SFs. We show that the best ML SF has a success rate of ~80% compared to ~70% for the best conventional SF when the goal is to find poses within RMSD of 1 Å from the native ones for 195 different protein-ligand complexes. Such a significant improvement (> 14%) in docking power will lead to better quality drug hits and ultimately help reduce costs associated with drug discovery.

We seek to advance structure-based drug design by designing SFs that significantly improve upon the protein-ligand modeling performance of conventional SFs. Our approach is to couple the modeling power of flexible machine learning algorithms with training datasets comprising hundreds of protein-ligand complexes with native poses of known high-resolution 3D crystal structures and experimentally-determined binding affinities. In addition, we computationally generate a large number of decoy poses and utilize their RMSD values from the native pose and a variety of features characterizing each complex. We compare the docking accuracies of several ML and existing conventional SFs of all three types, force-field, empirical, and knowledge-based, on diverse and independent test sets. We also perform a systematic analysis of the ability of the proposed SFs in identifying native poses of ligands that are docked to novel protein targets. Further, we assess the impact of training set size on the docking performance of the conventional BA-based SFs and the proposed RMSD-based models.

The remainder of the paper is organized as follows. The next section presents the compound database used for the comparative assessment of SFs, the physicochemical features extracted to characterize the compounds, the procedure for decoy generation and formation of training and test datasets, and conventional SFs and the ML methods that we employ. Then, we present results comparing the docking powers of conventional and ML SFs on diverse and homogeneous test sets. We also compare the performance of the ML techniques on novel drug targets and analyze how they are impacted by training set size. Finally, we summarize these results and conclude our work.

## Materials and methods

### Compound database

We used the 2007 version of PDBbind [8], the same complex database that Cheng et al. used as a benchmark in their recent comparative assessment of sixteen popular conventional SFs [2]. PDBbind is a selective compilation of the Protein Data Bank (PDB) database [19]. Both databases are publicly accessible and regularly updated. The PDB is periodically mined and only complexes that are suitable for drug discovery are filtered into the PDBbind database. In PDBbind, a number of filters are imposed to obtain high-quality protein-ligand complexes with both experimentally-determined BA and three-dimensional structure from PDB [2]. A total of 1300 protein-ligand complexes are compiled into a *refined set *after applying rigorous and systematic filtering criteria. The PDBbind curators compiled another list out of the refined set. It is called the *core set *and is mainly intended to be used for benchmarking docking and scoring systems. The core set is composed of diverse protein families and diverse binding affinities. BLAST [20] was employed to cluster the refined set based on protein sequence similarity with a 90% cutoff. From each resultant cluster, three protein-ligand complexes were selected to be its representatives in the core set. A cluster must fulfill the following criteria to be admitted into the core set: (i) it has at least four members and (ii) the BA of the highest-affinity complex must be at least 100-fold of that of the complex with the lowest one. The representatives were then chosen based on their BA rank: the complex having the highest rank, the middle one, and the one with the lowest rank. The approach of constructing the core set guarantees unbiased, reliable, and biochemically rich test set of complexes. In order to be consistent with the comparative framework used to assess the sixteen conventional SFs mentioned above [2], we too consider the 2007 version of PDB-bind which consists of a 1300-complex refined set and a 195-complex core set (with 65 clusters).

### Compound characterization

For each protein-ligand complex, we extracted physico-chemical features used in the empirical SFs X-Score [6] (a set of 6 features denoted by *X*) and AffiScore [21] (a set of 30 features denoted by *A*) and calculated by GOLD [10] (a set of 14 features denoted by *G*), and geometrical features used in the ML SF RF-Score [22] (a 36-feature set denoted by *R*). The software packages that calculate X-Score, AffiScore (from SLIDE), and RF-Score features were available to us in an open-source form from their authors and a full list of these features are provided in the appendix of [4]. The GOLD docking suite provides a utility that calculates a set of general descriptors for both molecules. The set includes some common ligand molecular properties such as: molecular weight, number of rotatable bonds, number of hydrogen bonds, solvent exposed descriptors, etc. Protein-specific features are also calculated that account for the number of polar, acceptor, and donatable atoms buried in the binding pocket. As a complex, two protein-ligand interaction features are calculated which are the number of ligand atoms forming H-bonds and the number of ligand atoms that clash with protein atoms. The full set of these features can be easily accessed and calculated via the *Descriptors *menu in GOLD.

### Decoy generation and formation of training and test sets

The training dataset derived from the 2007 refined set is referred to as the *primary training set *(1105 complexes) and we denote it by *Pr*. It is composed of the 1300 refined-set complexes of 2007, excluding those 195 complexes present in the core set (denoted by *Cr *) of the same year's version. The proteins of both these sets form complexes with ligands that were observed bound to them during 3D structure identification. These ligands are commonly known as native ligands and the conformation in which they were found at their respective binding sites are referred to as true or native poses. In order to assess the docking power of SFs in distinguishing true poses from random ones, a decoy set was generated for each protein-ligand complex in *Pr *and *Cr*. We utilize the decoy set produced for the core set *Cr *by Cheng et al. [2] using four popular docking tools: LigandFit in Discovery Studio, Surflex in SYBYL, FlexX in SYBYL (currently in LeadIT [23]), and GOLD. From each tool, a diverse set of binding poses was generated by controlling docking parameters as described in [2]. This process generated a total of ~2000 poses for each protein-ligand complex from the four docking protocols combined. Binding poses that are more than 10 Å away, in terms of RMSD (root-mean-square deviation), from the native pose are discarded. The remaining poses are then grouped into ten 1 Å bins based on their RMSD values from the native binding pose. Binding poses within each bin were further clustered into ten clusters based on their similarities [2]. From each such subcluster, the pose with the lowest noncovalent interaction energy with the protein was selected as a representative of that cluster and the remaining poses in that cluster were discarded. Therefore, at the end of this process, decoy sets consisting of (10 bins × 10 representatives =) 100 diverse poses were generated for each protein-ligand complex. Since we have access to the original *Cr *decoy set, we used it as is and we followed the same procedure to generate the decoy set for the training data *Pr*. Since we did not have access to Discovery Studio software, we did not use LigandFit protocol for the training data. In order to keep the size of the training set reasonable, we generated 50 decoys for each protein-ligand complex instead of 100 as it is the case for *Cr *complexes. Due to geometrical constraints during decoy generation, the final number of resultant decoys for some complexes does not add up exactly to 50 for *Pr *and 100 for *Cr*. It should be noted that the decoys in the training set are completely independent of those in the test set since both datasets share no ligands from which these decoys are generated.

As noted earlier, in the ligand docking problem with which we are concerned in this paper, the task is to identify the correct binding mode of a ligand from among a set of (computationally-generated) poses. The closer, in terms of RMSD, a pose is to the experimentally-determined native pose, the better [2]. We develop two types of ML SFs in this work to identify poses close to the native one. The first type are trained on training complexes with (known experimentally-determined) binding affinity (BA) as the response variable. To assess their docking accuracy, their predicted BA on a separate set of test complexes is used to distinguish promising poses from less promising ones. Note that for the test complexes, the experimentally-determined BA and actual RMSD values are not used during BA prediction; the actual RMSD value for test complexes is only used to assess docking accuracy. In all previous work, BA has been used for identifying near-native poses, which carries with it the implicit assumption that higher predicted BA implies lower RMSD for a pose. We believe a better approach is to model RMSD instead of BA. Therefore, the second set of SFs we build are trained on training complexes with (known) RMSD as the response variable. The accuracy of this approach, as in the case of BA-based SFs, is assessed on a separate set of test complexes by ranking poses according to predicted RMSD values: the lower the predicted RMSD, the more likely a pose is closer to the native pose. Note that for the test complexes the experimental BA and actual RMSD values are not used during RMSD prediction; the actual RMSD value is used only for docking accuracy assessment after prediction. RMSD-based SFs have three advantages over BA-based SFs. First, RMSD-based SFs model the same parameter (RMSD) that is used for pose ranking in-stead of relying on a related parameter (BA). Second, BA-based SFs are trained on experimental BA values, which are inherently noisy, whereas RMSD-based SFs use computationally-determined RMSD values during training which makes them less error prone. Third, during training, multiple decoys with different RMSD values can be computationally generated per complex. Therefore, the number of training records that can be utilized by RMSD-based SFs is the product of the number of different training complexes and the average number of computationally-generated poses per training complex. This training set size can be much larger compared to that available to BA-based SFs which is limited to as many training records as the number of different training complexes because BA values can be experimentally determined only for native poses, not for decoys. As it will be shown later, our novel RMSD-based approach provides significantly superior accuracy compared to conventional BA-based prediction.

For the two types of SFs, two versions of training and test data sets are created. The first version uses BA as the dependent variable (*Y *= BA) and the size of *Pr *remains fixed at 1,105 while *Cr *includes 16,554 complex conformations because it consists of native poses and a decoy set for each native pose. The dependent variable of the second version is RMSD (*Y *= RMSD) and because both training and test sets consist of native and decoy poses, the size of *Pr *expands to 39,085 while *Cr *still retains the same 16,554 complex conformations.

For all protein-ligand complexes, for both native poses and computationally-generated decoys, we extracted *X, A, R*, and *G *features. By considering all fifteen combinations of these four types of features (i.e., *X, A, R, G, X *∪ *A, X *∪ *R, X *∪ *G, A *∪ *R, A *∪ *G, R *∪ *G, X *∪ *A *∪ *R, X *∪ *A *∪ *G, X *∪ *R *∪ *G, A *∪ *R *∪ *G*, and *X *∪ *A *∪ *R *∪ *G*), we generated (15 × 2 =) 30 versions of the *Pr *and *Cr *data sets, which we distinguish by using the notation $Pr_{F}^{Y}$and $Cr_{F}^{Y}$to denote that the data set is characterized by the feature set *F *and its dependent variable is *Y *. For instance, $Pr_{XR}^{BA}$denotes the version of *Pr *comprising the set of features *X ∪ R *(referred to simply as XR) and experimentally-determined BA data for complexes in the *Pr *dataset.

### Conventional scoring sunctions

A total of sixteen popular conventional SFs are compared to ML SFs in this study. The sixteen functions are either used in mainstream commercial docking tools and/or have been developed in academia. The functions were recently compared against each other in a study conducted by Cheng et al. [2]. This set includes five SFs in the Discovery Studio software [12]: LigScore, PLP, PMF, Jain, and LUDI. Five SFs in SYBYL software [24]: D-Score, PMF-Score, G-Score, ChemScore, and F-Score. GOLD software [10] contributes three SFs: GoldScore, ChemScore, and ASP. GlideScore in the Schrödinger software [25]. Besides, two standalone scoring functions developed in academia are also assessed, namely, DrugScore [13] and X-Score [6]. Some of the SFs have several options or versions, these include LigScore (LigScore1 and LigScore2), PLP (PLP1 and PLP2), and LUDI (LUDI1, LUDI2, and LUDI3) in Discovery Studio; GlideScore (GlideScore-SP and GlideScore-XP) in the Schrödinger software; DrugScore (DrugScore-PDB and DrugScore-CSD); and X-Score (HPScore, HMScore, and HSScore). For brevity, we only report the version and/or option that yields the best performance on the PDBbind benchmark that was considered by Cheng et al.

### Machine learning methods

We utilize a total of six regression techniques in our study: multiple linear regression (MLR), multivariate adaptive regression splines (MARS), *k*-nearest neighbors (*k*NN), support vector machines (SVM), random forests (RF), and boosted regression trees (BRT) [26]. These techniques are implemented in the following R language packages that we use [27]: the package *stats *readily available in R for MLR, *earth *for MARS [28], *kknn *for *k*NN [29], *e1071 *for SVM [30], *randomForest *for RF [31], and *gbm *for BRT [32]. These methods benefit from some form of parameter tuning prior to their use in prediction. For example, the most important parameters in MARS are the number of terms (or basis functions) in the model, the *degree *of each term, and the *penalty *associated with adding new terms. Here we only tune the degree and penalty parameters and leave the final number of terms of MARS models to be automatically selected by the MARS algorithm implementation we use [28]. The *k*NN method has two parameters that require optimization: the neighborhood size *k *and the degree of Minkowski distance *q *[29]. For the SVM model, we have three parameters to optimize: the complexity constant *C*, the width of the ε-insensitive zone ε, and the width *σ *of the radial basis function that is used as a *kernel *[30]. RF algorithm has effectively only one important parameter *mtry *which determines the number of features to be randomly selected at each node split when growing the forest's trees [31]. The number of unpruned trees in the forest was fixed at 2000. BRT, on the other hand, has several parameters in addition to the most important two we tune: the *number of trees *and the *interaction depth *between the features [32]. The number of trees is optimized automatically using a cross-validation scheme internally implemented in the BRT algorithm [32]. The number of trees is tuned simultaneously with the interaction depth that controls their sizes. The shrinkage (or learning) rate of the BRT algorithm is set to 0.005 in all our experiments.

The values of the aforementioned parameters were selected so as to optimize the mean-squared errors on validation complexes sampled without replacement from the training set and independent of the test data. Out-of-bag instances were used as validation complexes to select the optimal value for the RF parameter *mtry*. Out-of-bag (OOB) refers to complexes that are not sampled from the training set when bootstrap sets are drawn to fit individual trees in RF models. The parameter values for MARS, *k*NN, SVM, and BRT were optimized by performing a grid search over a suitable range in conjunction with 10-fold cross-validation over the training set *Pr*. The resulting optimal parameter values are provided in Table 1. This optimization was done based on $Pr_{F}^{BA}$ for any given feature set *F *; optimizing based on $Pr_{F}^{RMSD}$ yielded similar parameter values, therefore, for brevity, we do not include them here. For every machine-learning method, we will be using these values to build ML SFs in the subsequent experiments.

**Table 1:** Optimal parameter values for MARS [28], *k*NN [29], SVM [30], RF [31], and BRT [32] models.

Model	Parameter	Feature set														
		
		X	A	R	G	XA	XR	XG	AR	AG	RG	XAR	XAG	XRG	ARG	XARG
MARS	*Degree*	2	1	1	1	1	1	1	1	1	1	1	1	1	1	1
	*Penalty*	2	6	5	6	7	2	6	7	6	5	6	7	6	5	6
*k*NN	*k*	15	13	14	16	9	19	17	19	18	17	18	19	17	18	19
	*q*	1	1	1	1	1	1	1	1	1	1	1	1	1	1	1
SVM	*C*	2	2	1	1	1	4	1	2	2	1	1	2	2	1	2
	ε	0.5	0	0.250	0.250	0.125	0.125	0.250	0.250	0.250	0.125	0.250	0.125	0.125	0.125	0.250
	*σ*	1	0.25	0.125	0.250	0.250	0.031	0.031	0.031	0.125	0.031	0.125	0.031	0.031	0.031	0.031
RF	*mtry*	3	18	8	7	31	5	8	10	16	17	14	20	21	25	35
BRT	*Interaction depth*	15	17	18	16	19	15	18	19	17	16	16	20	18	17	20
	*Number of trees*	1114	1523	1573	1208	1371	2113	1610	2950	2181	2303	2213	2590	2854	2921	2859

## Results and discussion

### Evaluation of scoring functions

In contrast to our earlier work in improving and examining scoring and ranking accuracies of different families of SFs [3,4], this study is devoted to enhancing and comparing SFs in terms of their docking powers. Docking power measures the ability of an SF to distinguish a promising binding mode from a less promising one. Typically, generated conformations are ranked in non-ascending order according to their predicted binding affinity (BA). Ligand poses that are very close to the experimentally-determined ones should be ranked high. Closeness is measured in terms of RMSD (in Å) from the true binding pose. Generally, in docking, a pose whose RMSD is within 2 Å from the true pose is considered a success or a hit.

In this work, we use comparison criteria similar to those used by Cheng et al. to compare the docking accuracies of sixteen popular conventional SFs. Doing so ensures fair comparison of ML SFs to those examined in that study in which each SF was assessed in terms of its ability to find the pose that is closest to the native one. More specifically, docking ability is expressed in terms of a success rate statistic *S *that accounts for the percentage of times an SF is able to find a pose whose RMSD is within a predefined cutoff value *C Å *by only considering the *N *topmost poses ranked by their predicted scores. Since success rates for various *C *(e.g., 0, 1, 2, and 3 Å) and *N *(e.g., 1, 2, 3, and 5) values are reported in this study, we use the notation $S_{C}^{N}$to distinguish between these different statistics. For example, $S_{1}^{2}$ is the percentage of protein-ligand complexes whose either one of the two best scoring poses are within 1 Å from the true pose of a given complex. It should be noted that $S_{0}^{1}$ is the most stringent docking measure in which an SF is considered successful only if the best scoring pose is the native pose. By the same token and based on the *C *and *N *values listed earlier, the least strict docking performance statistic is $S_{3}^{5}$ in which an SF is considered successful if at least one of the five best scoring poses is within 3 Å from the true pose.

### ML vs. conventional approaches on a diverse test set

After building six ML SFs, we compare their docking performance to the sixteen conventional SFs on the core test *Cr *that comprises thousands of protein ligand complex conformations corresponding to 195 different native poses in 65 diverse protein families. As mentioned earlier, we conducted two experiments. In the first, BA values predicted using the conventional and ML SFs were used to rank poses in a non-ascending order for each complex in *Cr*. In the other experiment, RMSD-based ML models directly predicted RMSD values that are used to rank in non-descending order the poses for the given complex.

**Figure 2:**
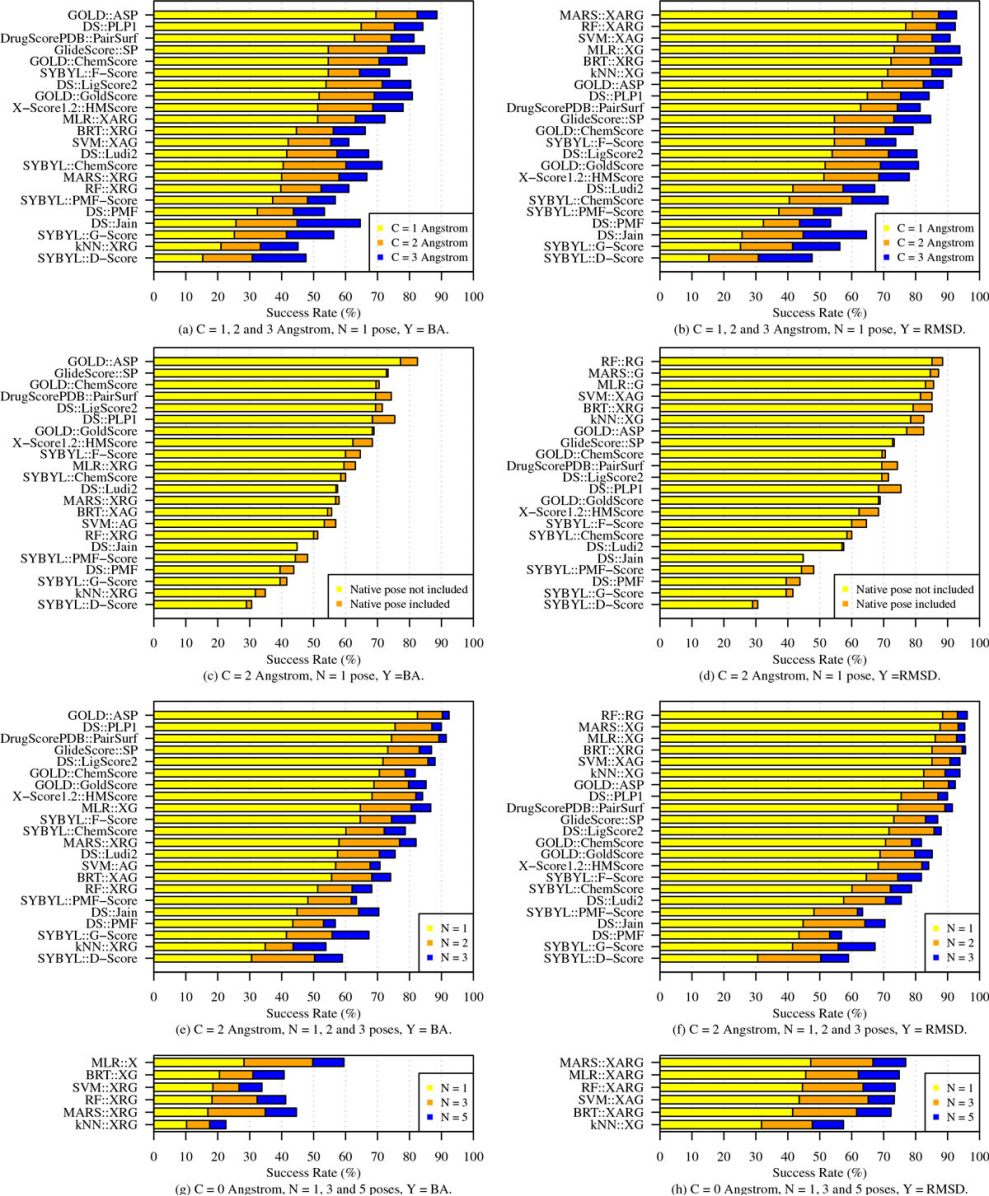
**Success rates of conventional and ML SFs in identifying binding poses that are closest to native ones**. The results show these rates by examining the top *N *scoring ligands that lie within an RMSD cut-off of *C *Å from their respective native poses. Panels on the left show success rates when binding-affinity based (*BA*) scoring is used and the ones on the right show the same results when ML SFs predicted *RMSD *values directly. Scoring of conventional SFs is BA-based in all cases and for comparison convenience we show their performance in the right panels as well.

By examining the true RMSD values of the best *N *scoring ligands using the two prediction approaches, success rates of SFs are computed; these are shown in Figure 2. Panels (a) and (b) in the figure show the success rates $S_{1}^{1}$, $S_{2}^{1}$, and $S_{3}^{1}$ for all 22 SFs. The SFs, as in the other panels, are sorted in non-ascending order from the most stringent docking test statistic value to the least stringent one. In the top two panels, for example, success rates are ranked based on $S_{1}^{1}$, then $S_{2}^{1}$ in case of a tie in $S_{1}^{1}$, and finally $S_{3}^{1}$ if two or more SFs tie in $S_{2}^{1}$. In both BA- and RMSD-based scoring, we find that the 22 SFs vary significantly in their docking performance. The top three BA-based SFs, GOLD::ASP, DS::PLP1, and DrugScorePDB::PairSurf, have success rates of more than 60% in terms of $S_{1}^{1}$ measure. That is in comparison to the BA-based ML SFs, the best of which has an $S_{1}^{1}$ value barely exceeding 50% (Figure 2(a)). On the other hand, the other six ML SFs that directly predict RMSD values achieve success rates of over 70% as shown in Figure 2(b). The top performing of these ML SFs, MARS::XARG, has a success rate of ~80%. This is a significant improvement (> 14%) over the best conventional SF, the empirical GOLD::ASP, whose $S_{1}^{1}$ value is ~70%. Similar conclusions can also be made for the less stringent docking performance measures $S_{2}^{1}$ and $S_{3}^{1}$ in which the RMSD cut-off constraint is relaxed to 2 Å and 3 Å, respectively.

The success rates plotted in the top two panels (Figure 2(a) and 2(b)) are reported when native poses are included in the decoy sets. Panels (c) and (d) of the same figure show the impact of removing the native poses on docking success rates of all SFs. It is clear that the performance of almost all SFs does not radically decrease by examining the difference in their $S_{2}^{1}$ statistics which ranges from 0 to ~5%. This, as it was noted by Cheng et al. [2], is due to the fact that some of the poses in the decoy sets are actually very close to the native ones. As a result, the impact of allowing native poses in the decoy sets is insignificant in most cases and therefore we include such poses in all other tests in the paper.

In reality, more than one pose is usually used from the outcomes of a docking run in the next stages of drug design for further experimentation. It is useful therefore to assess docking accuracy of SFs when more than one pose is considered (i.e., *N *> 1). Figure 2(e) and 2(f) show the success rates of SFs when the RMSD values of the best 1, 2, and 3 scoring poses are examined. These rates correspond, respectively, to $S_{1}^{2}$, $S_{2}^{2}$, and $S_{3}^{2}$. The plots show a significant boost in performance for almost all SFs. By comparing $S_{1}^{2}$ to $S_{3}^{2}$, we observe a jump in accuracy from 82% to 92% for GOLD::ASP and from 87% to 96% for RF::RG that models RMSD values directly. Such results signify the importance of examining an ensemble of top scoring poses because there is a very good chance it contains relevant conformations and hence good drug candidates.

Upon developing RMSD-based ML scoring models, we noticed excellent improvement over their binding-affinity-based counterparts as shown in Figure 2. We conducted an experiment to investigate whether they will maintain a similar level of accuracy when ML SFs are examined for their ability to pinpoint the native poses from their respective 100-pose decoy sets. The bottom two panels, (g) and (h), plot the success rates in terms of $S_{0}^{1}$, $S_{0}^{3}$, and $S_{0}^{5}$ for the six ML SFs. By examining the five best scoring poses, we notice that the top BA-based SF, MLR::X, was able to distinguish native binding poses in ~60% of the 195 decoy sets whereas the top RMSD-based SF, MARS::XARG, achieved a success rate of $S_{0}^{5}$ = 77% on the same protein-ligand complexes. It should be noted that both sets of ML SFs, the BA- and RMSD-based, were trained and tested on completely disjoint test sets. Therefore, this gap in performance is largely due to the explicit modeling of RMSD values and the corresponding abundance of training data which includes information from both native and computationally-generated poses.

### ML vs. conventional approaches on homogeneous test sets

**Figure 3:**
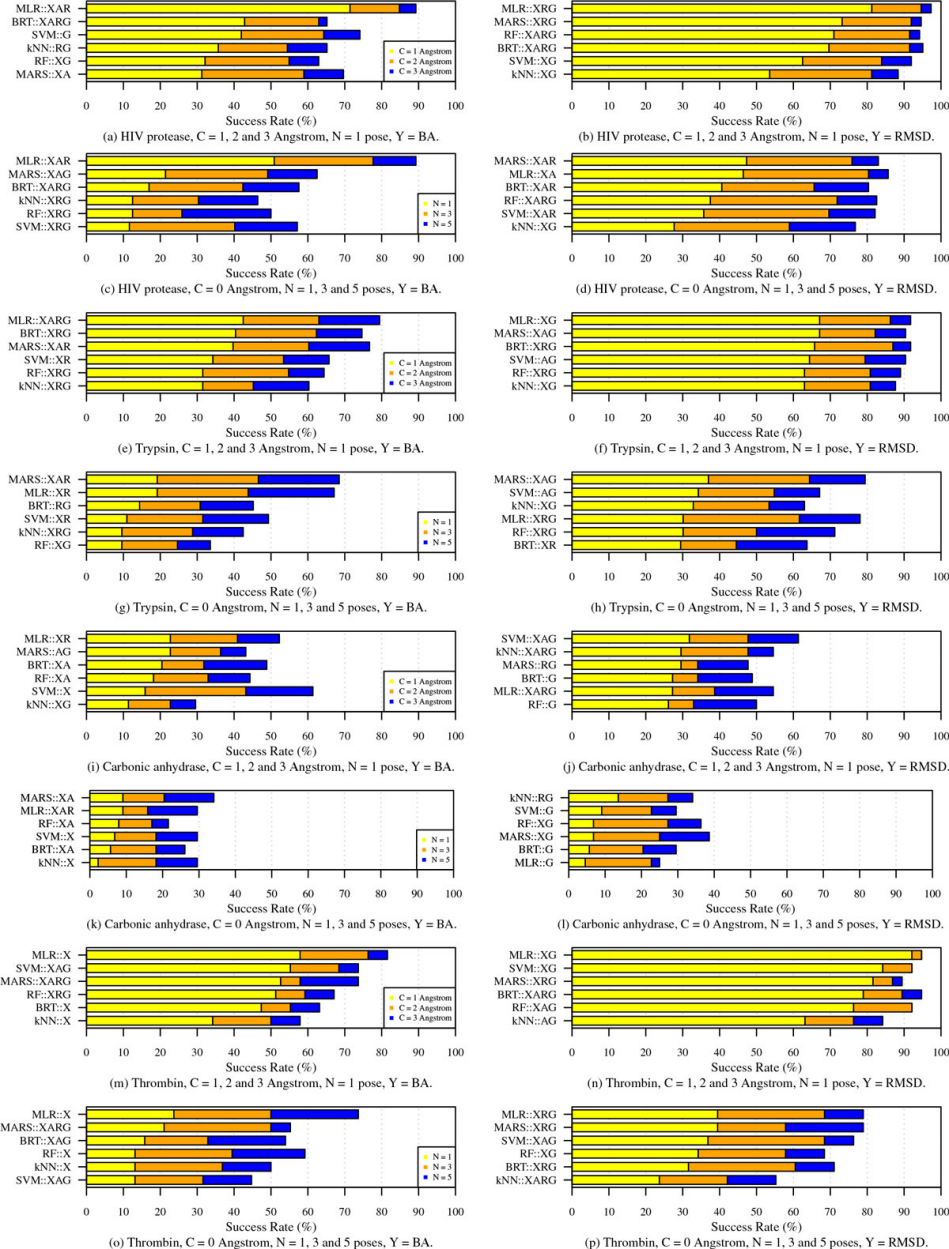
**Success rates of ML SFs in identifying binding poses that are closest to native ones observed in four protein families: HIV protease (a-d), trypsin (e-h), carbonic anhydrase (i-l), and thrombin (m-p)**. The results show these rates by examining the top *N *scoring ligands that lie within an RMSD cut-off of *C *Å from their respective native poses. Panels on the left show success rates when binding-affinity based (*BA*) scoring is used and the ones on the right show the same results when ML SFs predicted *RMSD *values directly.

In the previous section, performance of SFs was assessed on the diverse test set *Cr*. The core set consists of more than sixty different protein families each of which is related to a subset of protein families in *Pr*. That is, while the training and test set complexes were different (at least for all the ML SFs), proteins present in the core test set were also present in the training set, albeit bound to different ligands. A much more stringent test of SFs is their evaluation on a completely new protein, i.e., when test set complexes all feature a given protein - test set is homogeneous - and training set complexes do not feature that protein. To address this issue, four homogeneous test sets were constructed corresponding to the four most frequently occurring proteins in our data: HIV protease (112 complexes), trypsin (73), carbonic anhydrase (44), and thrombin (38). Each of these protein-specic test sets was formed by extracting complexes containing the protein from *Cr *(one cluster or three complexes) and *Pr *(remaining complexes). For each test set, we retrained BRT, RF, SVM, *k*NN, MARS, and MLR models on the non-test set complexes of *Pr*. Figure 3 shows the docking performance of resultant BA and RMSD-based ML scoring models on the four protein families. The plots clearly show that success rates of SFs are dependent on the protein family under investigation. It is easier for some SFs to distinguish good poses for HIV protease and thrombin than for carbonic anhydrase. The best performing SFs on HIV protease and thrombin complexes, performance of resultant BA and RMSD-based ML scoring models on the four protein families. The plots clearly show that success rates of SFs are dependent on the protein family under investigation. It is easier for some SFs to distinguish good poses for HIV protease and thrombin than for carbonic anhydrase. The best performing SFs on HIV protease and thrombin complexes, MLR::XRG and MLR::XG, respectively, achieve success rates of over 95% in terms of $S_{1}^{3}$ as shown in panels (b) and (n), whereas no SF exceeded 65% in success rate in case of carbonic anhydrase as demonstrated in panels (i) and (j). Finding the native poses is even more challenging for all SFs, although we can notice that RMSD-based SFs outperform those models that rank poses using predicted BA. The exception to this is the SF MLR::XAR whose performance exceeds all RMSD-based ML models in terms of the success rate in reproducing native poses as illustrated in panels (c) and (d).

The results also indicate that multivariate linear regression models (MLR), which are basically empirical SFs, are the most accurate across the four families, whereas ensemble learning models, RF and BRT, unlike their good performance in Figure 2, appear to be inferior compared to simpler models in Figure 3. This can be attributed to the high rigidity of linear models compared to ensemble approaches. In other words, linear models are not as sensitive as ensemble techniques to the presence or absence of certain protein family in the data on which they are trained. On the other hand, RF- and BRT-based SFs are more flexible and adaptive to their training data that in some cases fail to generalize well enough to completely different test proteins as seen in Figure 3. In practice, however, it has been observed that more than 92% of today's drug targets are similar to known proteins in PDB [33], an archive of high quality complexes from which our training and test compounds originated. Therefore, if the goal of a docking run is to identify the most stable poses, it is important to consider sophisticated SFs (such as RF and BRT) calibrated with training sets containing some known binders to the target of interest. Simpler models, such as MLR and MARS, tend to be more accurate when docking to novel proteins that are not present in training data.

Sophisticated ML algorithms are not the only critical element in building a capable SF. Features to which they are fitted also play an important role as can be seen in Figure 3. By comparing the right panels to the ones on the left, we can notice that X-Score features (X) are almost always present in BA-based SFs while those provided by GOLD (G) are used more to model RMSD explicitly. This implies that X-Score features are more accurate than other feature sets in predicting BA, while GOLD features are the best for estimating RMSD and hence poses close to the native one.

### Performance of ML SFs on novel targets

The training-test set pair (*Pr, Cr *) is a useful benchmark when the aim is to evaluate the performance of SFs on targets that have some degree of sequence similarity with at least one protein present in the complexes of the training set. This is typically the case since, as it was mentioned earlier, 92% of drug targets are similar to known proteins [33]. When the goal is to assess SFs in the context of novel protein targets, however, the training-test set pair (*Pr, Cr *) is not that suitable because of the partial overlap in protein families between *Pr *and *Cr*. We considered this issue to some extent in the previous section, where we investigated the docking accuracy of SFs on four different protein-specific test sets after training them on complexes that did not have the protein under consideration. This resulted in a drop in performance of all SFs, especially, in the case of carbonic anhydrase as a target. However, even if there are no common proteins between training and test set complexes, different proteins at their binding sites may have sequence and structural similarities, which influence docking results. To more rigorously and systematically assess the performance of BA and RMSD-based ML SFs on novel targets, we performed a separate set of experiments in which we limited BLAST sequence similarity between the binding sites of proteins present in the training and test set complexes. Sequence similarity was used to construct the core test set and it was also noted by Ballester and Mitchell as being relevant to testing the efficacy of SFs on a novel target [34].

Specifically, for each similarity cut-off value *S *= 30%, 40%, 50%,..., 100%, we constructed 100 different independent 100-complex test and *T*-complex training set pairs. Two versions were created out of these training and test set pairs. The first version uses BA as a response variable that SFs will be fitted to, predict, and employ to assess poses. The response variable of the other version is the RMSD value of true poses (RMSD = 0 Å) and computer generated decoys (with RMSD > 0 Å) of each original protein-ligand complex in every training and test dataset pair. A total of 20 poses per complex have been used in this second version. Then, we trained BA and RMSD scoring models (MLR, MARS, *k*NN, SVM, RF, and BRT) using XARG features on the training set and evaluated them on the corresponding test set, and determined their average performance over the 100 training-test-set pairs to obtain robust results. Since SF docking performance depends upon both similarity cut-off and training set size and since training set size is constrained by similarity cut-off (a larger *S *means a larger feasible *T *), we investigated different ranges of *S *(30% to 100%, 50% to 100%, and 70% to 100%) and for each range we set *T *close to the largest feasible value for the smallest *S *value in that range. Each test and training set pair was constructed as follows. We randomly sampled a test set of 100 protein-ligand complexes without replacement from all complexes at our disposal: 1105 in *Pr *+ 195 in *Cr *= 1300 complexes. The remaining 1200 complexes were randomly scanned until *T *different complexes were found that had protein binding site similarity of *S *% or less with the protein binding sites of all complexes in the test set - if less than *T *such complexes were found, then the process was repeated with a new 100-complex test set.

**Figure 4:**
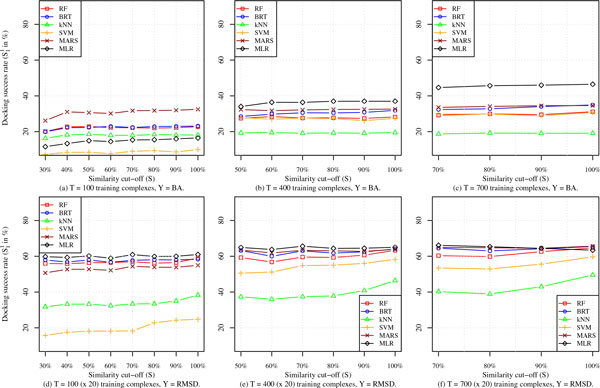
**Performance of SFs in terms of docking success rate $S_{1}^{1}$ as a function of BLAST sequence similarity cutoff between binding sites of proteins in training and test complexes**. In panels (a)-(c), a single (native) pose is used per training complex, whereas in panels (d)-(f) 20 randomly-selected poses are used per training complex.

The performance of the six scoring models in terms of their $S_{1}^{1}$ docking accuracy is depicted in Figure 4 for various similarity cut-offs and training set sizes. The plots in each column (a) and (d), (b) and (e), and (c) and (f) of Figure 4) show docking power results for similarity cut-offs of 30% to 100%, 50% to 100%, and 70% to 100% for which *T *= 100, 400, and 700 complexes is the largest training set size feasible for *S *= 30%, 50%, and 70%, respectively. The results in these plots are consistent with those obtained for the four protein families presented in the previous section and illustrated in Figure 3. More specifically, we notice that simpler models such as MLR::XARG and MARS::XARG perform the best across almost all values of similarity cut-offs (*S *= 30%, 50%, or 70% - 100%), training set sizes (*T *= 100, 400, or 700 complexes), and response variables (*Y *= BA or RMSD). This is mainly due to their rigidity. The performance of such models do not suffer as much as the more flexible ML SFs when their training and test proteins have low sequence similarity. On the other hand, the SFs based on MLR and MARS are also less responsive to increasing the similarity between protein families in the training and test sets. Unlike the other four nonlinear ML SFs, we can observe that the performance curves of MLR and MARS are flat and do not seem to react to having more and more similar training and test proteins. This observation is more clear in the bottom row of plots of Figure 4 where the training set sizes are large enough (i.e., 2000 ligand poses or more). Plot (f) shows that the RMSD-based SFs RF and BRT are catching up with MLR and MARS and can eventually surpass them in terms of performance as training set sizes become larger. Similar to RF and BRT, the other nonlinear RMSD SFs, namely *k*NN and SVM, have the sharpest increase in docking performance as similarity cut-off *S *increases. However, unlike the ensemble SFs RF and BRT, *k*NN and SVM SFs are the least reliable models when ligand poses need to be scored for novel targets.

To summarize, imposing a sequence similarity cut-off between the binding sites of proteins in training and test set complexes has an expected adverse impact on the accuracy of all scoring models. However, increasing the number of training complexes helps improve accuracy for all similarity cut-offs as we will show in more detail in the next section. Scoring functions based on MLR and MARS have the best accuracy when training set sizes are small which is typically the case when the response variable is binding affinity. The other generally-competitive ML models are RF and BRT whose accuracies surpass all other SFs when evaluated on targets that have some sequence similarity with their training proteins.

### Impact of training set size

An important factor influencing the accuracy of ML SFs is the size of the training dataset. In the case of BA-based ML SFs, training dataset size can be increased by training on a larger set of protein-ligand complexes with known binding affinity values. In the case of RMSD-based SFs, on the other hand, training dataset size can be increased not only by considering a large number of protein-ligand complexes in the training set, but also by using a larger number of computationally-generated ligand poses per complex since each pose provides a new training record because it corresponds to a different combination of features and/or RMSD value. Unlike experimental binding affinity values, which have inherent noise and require additional resources to obtain, RMSD from the native conformation for a new ligand pose is computationally determined and is accurate.

**Figure 5:**
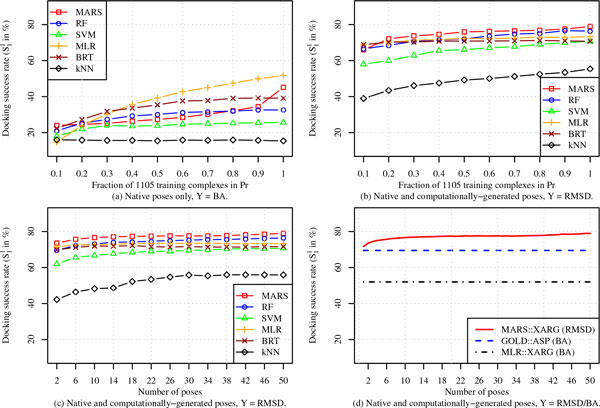
**Dependence of docking accuracy of ML scoring models on training set size when training complexes are selected randomly (without replacement) from *Pr *and the models are tested on *Cr***. The size of the training data was increased by including more protein-ligand complexes (a) and (b) or more computationally-generated poses for all complexes (c) and (d).

We carried out three different experiments to determine: (i) the response of BA-based ML SFs to increasing number of training protein-ligand complexes, (ii) the response of RMSD-based ML SFs to increasing number of training protein-ligand complexes while the number of poses for each complex is fixed at 50, and (iii) the response of RMSD-based ML SFs to increasing number of computationally-generated poses while the number of protein-ligand complexes is fixed at 1105. In the first two experiments, we built 6 ML SFs, each of which was trained on a randomly sampled *x*% of the 1105 protein-ligand complexes in *Pr*, where *x *= 10, 20,..., 100. The dependent variable in the first experiment is binding affinity (*Y *= BA), and the performance of these BA-based ML SFs is shown in Figure 4(a) and partly in Figure 4(d) (MLR::XARG). The set of RMSD values from the native pose is used as a dependent variable for ML SFs trained in the second experiment (*Y *= RMSD). For a given value of *x*, the number of conformations is fixed at 50 ligand poses for each protein-ligand complex. The docking accuracy of these RMSD-based ML models is shown in Figure 5(b). In the third experiment, all 1105 complexes in *Pr *were used for training the RMSD-based ML SFs (i.e., *Y *= RMSD) with *x *randomly sampled poses considered per complex, where *x *= 2, 6, 10,..., 50; results for this are reported in Figure 5(c) and partly in Figure 5(d) (MARS::XARG). In all three experiments, results reported are the average of 50 random runs in order to ensure all complexes and a variety of poses are equally represented. All training and test complexes in these experiments are characterized by the XARG (=*X *∪ *A *∪ *R *∪ *G*) features.

From Figure 5(a), it is evident that increasing training dataset size has a positive impact on docking accuracy (measured in terms of $S_{1}^{1}$ success rate), although it is most appreciable in the case of MLR::XARG and MARS::XARG, two of the simpler models, MLR being linear and MARS being piecewise linear. The performance of the other models, which are all highly nonlinear, seems to saturate at 60% of the maximum training dataset size used. The performance of all six models is quite modest, with MLR::XARG being the only one with docking success rate (slightly) in excess of 50%. The explanation for these results is that binding affinity is not a very good response variable to learn for the docking problem because the models are trained only on native poses (for which binding affinity data is available) although they need to be able to distinguish between native and non-native poses during testing. This means that the training data is not particularly well suited for the task for which these models are used. An additional reason is that experimental binding affinity data, though useful, is inherently noisy. The flexible highly nonlinear models, RF, BRT, SVM, and *k*NN, are susceptible to this noise because the training dataset (arising only from native poses) is not particularly relevant to the test scenario (consisting of both native and non-native poses). Therefore, the more rigid MLR and MARS models fair better in this case.

When RMSD is used as the response variable, the training set consists of data from both native and nonnative poses and hence is more relevant to the test scenario and the RMSD values, being computationally determined, are also accurate. Consequently, docking accuracy of all SFs improves dramatically compared to their BA-based counterparts as can be observed by comparing Figure 5(a) to Figure 5(b) and 5(c). We also notice that all SFs respond favorably to increasing training set size by either considering more training complexes (Figure 5(b)) or more computationally-generated training poses (Figure 5(c)). Even for the smallest training set sizes in Figure 5(b) and 5(c), we notice that the docking accuracy of most RMSD-based SFs is about 70% or more, which is far better than the roughly 50% success rate for the largest training set size for the best BA-based SF MLR::XARG.

In Figure 5(d), we compare the top performing RMSD SF, MARS::XARG, to the best BA-based SFs, GOLD::ASP and MLR::XARG, to show how docking performance can be improved by just increasing the number of computationally-generated poses, an important feature that RMSD-based SFs possess but which is lacking in their BA-based conventional counterparts. To increase the performance of these BA-based SFs to a comparable level, thousands of protein-ligand complexes with high-quality experimentally-determined binding affinity data need to be collected. Such a requirement is too expensive to meet in practice. Furthermore, RMSD-based SFs with the same training complexes will still likely outperform BA-based SFs.

### Impact of the type and number of features

The binding pose of a protein-ligand complex depends on many physicochemical interaction factors that are too complex to be accurately captured by any one approach. Therefore, we perform two different experiments to investigate how utilizing different types of features from different scoring tools, X-Score, AffiScore, RF-Score, and GOLD, and considering an increasing number of features affects the performance of the various ML models. In the first experiment, the ML models were trained on *Pr *characterized by all 15 combinations of X, A, R, and G feature types and tested on the corresponding core test *Cr *characterized by the same features. Table 2 reports the $S_{1}^{1}$ docking success rate for three groups of ML SFs. The first set (Table 2 top part) of 90 (6 methods × 15 feature combinations) BA-based SFs is trained on 1105 *Pr *complexes. The second set (Table 2 middle part) of 90 RMSD-based SFs is again trained on the 1105 *Pr *complexes with one randomly sampled pose from 50 poses generated per complex. Therefore, the training set size for these first two groups of SFs is identical and consists of 1105 training records, with the only difference being the response variable that they are trained for, BA in the first case and RMSD in the second case. The final (Table 2 bottom part) 90 RMSD-based SFs are trained on 1105 *Pr *complexes, with 50 poses per complex, so that its training set size is 1105 × 50 = 55,250 records.

**Table 2:** Docking success rate $S_{1}^{1}$ (in %) of ML SFs trained on *Pr *and tested on *Cr *complexes when characterized by different features.

Y (T)	Model	Feature set															
		
		X	A	R	G	XA	XR	XG	AR	AG	RG	XAR	XAG	XRG	ARG	XARG	Average
BA (1105)	MARS	28.72	20.00	5.13	18.46	34.36	36.92	28.21	21.03	16.41	8.21	37.95	28.72	**40.00**	8.21	36.41	24.58
	RF	23.08	23.08	10.77	24.10	30.52	30.77	37.44	21.54	30.00	25.65	31.03	34.12	**39.75**	25.90	32.31	28.00
	SVM	26.67	30.26	4.62	19.49	30.77	23.08	29.23	20.51	41.54	30.26	26.15	**42.05**	37.44	38.97	41.54	29.51
	MLR	43.08	18.97	9.74	28.21	33.33	46.67	47.18	21.03	40.00	33.85	46.15	49.23	49.74	40.00	**51.28**	**37.23**
	BRT	23.85	25.39	9.24	39.23	30.52	30.26	42.31	22.82	37.69	33.85	33.85	44.36	**44.62**	36.67	43.08	33.18
	*k*NN	17.95	11.28	8.72	16.41	9.23	18.46	15.90	11.28	15.38	17.95	15.38	17.44	**21.03**	14.36	17.44	15.21
	Average	27.22	21.50	8.04	24.32	28.12	31.03	33.38	19.71	30.17	24.96	31.75	35.99	**38.76**	27.35	37.01	27.95

RMSD (1105)	MARS	45.64	42.05	26.15	72.31	54.87	52.31	73.85	33.85	70.26	71.28	52.82	70.26	72.82	67.18	**75.90**	**58.77**
	RF	32.72	32.31	13.85	67.39	39.80	42.26	70.36	32.10	64.82	68.10	41.95	64.82	**72.00**	64.51	67.18	51.61
	SVM	29.74	28.72	6.15	66.15	37.95	30.77	**70.77**	32.31	61.54	56.41	45.64	64.10	62.05	58.97	63.08	47.62
	MLR	44.62	35.90	6.15	70.26	58.46	54.36	72.31	40.51	70.26	65.64	56.92	**75.90**	70.26	67.69	69.23	57.23
	BRT	38.77	34.87	8.10	70.98	52.00	41.13	**74.77**	35.59	67.70	69.02	48.92	71.39	72.51	65.33	67.49	54.57
	*k*NN	31.79	24.10	3.08	55.38	30.77	17.44	**62.56**	15.90	40.00	38.97	22.05	44.62	45.13	37.95	41.03	34.05
	Average	37.21	32.99	10.58	67.08	45.64	39.71	**70.77**	31.71	62.43	61.57	44.72	65.18	65.79	60.27	63.98	50.64

RMSD (1105x50)	MARS	44.10	31.79	5.13	72.82	62.56	49.74	75.38	34.87	73.33	70.77	64.10	76.92	72.31	72.31	**78.97**	59.01
	RF	39.39	48.10	22.46	70.77	61.95	54.77	72.41	50.97	73.64	75.18	63.79	76.00	75.49	75.28	**76.90**	**62.47**
	SVM	36.41	43.08	10.26	66.15	54.87	43.08	70.77	43.08	71.79	65.13	57.95	**74.36**	72.31	69.74	70.77	56.65
	MLR	45.64	36.41	6.15	70.26	56.92	52.31	**73.34**	37.95	71.79	71.28	57.44	72.82	71.79	70.77	73.33	57.88
	BRT	46.15	36.92	13.33	71.59	54.36	54.87	71.59	42.56	70.77	70.77	56.92	70.26	**72.31**	71.28	71.28	58.33
	*k*NN	36.41	46.15	23.59	61.03	51.28	41.03	**71.28**	45.13	60.00	53.33	49.23	61.54	60.51	53.33	55.90	51.32
	Average	41.35	40.41	13.49	68.77	57.01	49.30	**72.46**	42.43	70.22	67.69	58.24	72.05	70.68	69.23	70.63	57.61

We notice that the $S_{1}^{1}$ value of almost all models improves by considering more than one type of feature rather than just X, A, R, or G features alone. The table also shows that RMSD SFs are substantially more accurate than their BA counterparts for each feature type and ML method. By comparing the 180 RMSD SFs with the corresponding 90 BA SFs across all feature types and ML models, we find that the former are, on average, almost twice as accurate as the BA approaches (50.64% and 57.61% vs. 27.95% -see Table 2 rightmost column). In terms of feature types, we note that the most accurate SFs always include X-Score and GOLD features. SFs that are fitted to the individual × and G features only are more accurate than their A and R counterparts whether they are BA or RMSD models. By averaging the performance of all ML models across all feature types, we see that the simple linear approach MLR outperforms other more sophisticated ML SFs that are trained to predict binding affinity. MARS outperforms all other RMSD SFs that are trained on the same number of training records (1105) as their BA counterparts. The lower part of the table shows that the ensemble SF RF that predicts the binding pose directly has the highest docking accuracy (62.47%), on the average, across 15 different feature types and MARS::XARG has the highest docking accuracy (78.97%) overall. Comparing the two versions of RMSD SFs in the middle and lower portions of the table, we notice that the largest gainers from increasing training set size are the most nonlinear ML techniques (RF, BRT, SVM and *k*NN). The results of Table 2 are useful in assessing the relative benefit of different types of features for the various ML models.

**Figure 6:**
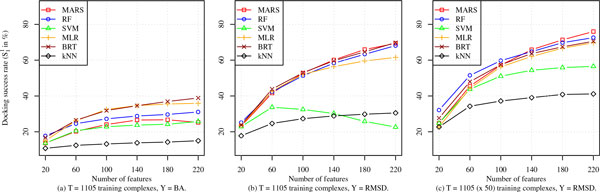
**Dependence of docking accuracy of ML scoring models on the number of features, with the features drawn randomly (without replacement) from a pool of X, A, R, and G-type features and used to train the ML models on the *Pr *dataset and then tested on the disjoint core set *Cr ***. In panels (a) and (b), a single pose (native pose in (a) and randomly-selected pose in (b) is used per training complex, whereas in panel (c) 50 randomly-selected poses are used per training complex.

A pertinent issue when considering a variety of features is how well different SF models exploit an increasing number of features. The features we consider are the X, A, G, and a larger set of geometrical features than the R feature set available from the RF-Score tool. Recall from the Compound Characterization subsection that RF-Score counts the number of occurrences of 36 different protein-ligand atom pairs within a distance of 12 Å. In order to have more features of this kind for this experiment, we produce 36 such counts for five contiguous distance intervals of 4 Å each: (0 Å, 4 Å], (4 Å, 8 Å],..., (16 Å, 20 Å]. This provides us 6 X, 30 A, 14 G, and (36 × 5 =) 180 geometrical features or a total of 220 features. We randomly select (without replacement) *x *features from this pool, where *x *= 20, 60, 100,..., 220, and use them to characterize the *Pr *dataset, which we then use to train the six ML models. These models are subsequently tested on the *Cr *dataset characterized by the same features. This process is repeated 100 times to obtain robust average $S_{1}^{1}$ statistics, which are plotted in Figure 6.

The performance of the BA SFs is depicted in Figure 6(a) whereas panels (b) and (c) of the same figure show the docking success rates for the RMSD versions of the scoring models. In order to fairly compare the docking performance of BA and RMSD SFs as number of features increase, we fixed their training set sizes to 1105 complexes as shown in Figure 6(a) and 6(b). We also show in Figure 6(c) the effect of increasing number of features on the docking performance of RMSD SFs when trained on all *Pr *complexes, with 50 poses per complex. The plots clearly indicate that RMSD SFs benefit the most from characterizing complexes with more descriptors. This is the case regardless of the number of records used to train RMSD SFs (compare plots (b) and (c) in Figure 6). The only exception is the RMSD SF based on SVM where it appears to overfit the 1105 training records when they are characterized by more than 60 features. This ML scoring function, however, performs better when trained on larger number of records and shows a slight increase in performance as more features are included in building the model. Other RMSD SFs such as RF, BRT, MLR, and MARS have much sharper slopes than SVM and *k*NN. Compare these SFs to their BA counterparts in Figure 6(a) where most of them show none to little improvement as the number of features increases due to overfitting. Not only are they resilient to overfitting, most RMSD SFs improve dramatically by extracting more relevant features. Adding more features may result in highest gains in performance when more training complexes are included as was discussed in the previous subsection.

## Conclusion

We found that ML models trained to explicitly predict RMSD values significantly outperform all conventional SFs in almost all testing scenarios. The estimated RMSD values of such models have a correlation coefficient of 0.7 on average with the true RMSD values. On the other hand, predicted binding affinities have a correlation of as low as -0.2 with the measured RMSD values. This difference in correlation explains the wide gap in docking performance between the top SFs of the two approaches. The empirical SF GOLD::ASP, which is the best conventional model, achieved a success rate of 70% in identifying a pose that lies within 1 Å from the native pose of 195 different complexes. On other hand, our top RMSD-based SF, MARS::XARG, has a success rate of ~80% on the same test set, which represents a significant improvement in docking performance. The linear ML SF, MLR::XARG, and its nonlinear extension, MARS::XARG, may be employed when the target is a protein not present in the training dataset used to build the scoring model. Ensemble SFs, however, may prove more reliable when there is some similarity between training set proteins and the target protein. We also observed steady gains in the performance of RMSD-based ML SFs as the training set size and number of features were increased by considering more descriptors and protein-ligand complexes and/or more computationally-generated ligand poses for each complex.

## Competing interests

The authors declare that they have no competing interests.

## Authors' contributions

Devised the comparison techniques and experiments: N.M. and H.A.

Implemented the techniques and carried out the experiments: H.A.

Analyzed the results: N.M. and H.A. Wrote the paper and revised it: H.A. and N.M.

## Declarations

The publication costs for this article were sourced from the National Science Foundation under Grant No. 1117900.

This article has been published as part of *BMC Bioinformatics *Volume 16 Supplement 6, 2015: Selected articles from the 10th International Meeting on Computational Intelligence Methods for Bioinformatics and Biostatistics. The full contents of the supplement are available online at http://www.biomedcentral.com/bmcbioinformatics/supplements/16/S6.
